# Joint effects of indoor air pollution and maternal psychosocial factors during pregnancy on trajectories of early childhood psychopathology

**DOI:** 10.1093/aje/kwae046

**Published:** 2024-04-17

**Authors:** Grace M Christensen, Michele Marcus, Aneesa Vanker, Stephanie M Eick, Susan Malcolm-Smith, Shakira F Suglia, Howard H Chang, Heather J Zar, Dan J Stein, Anke Hüls

**Affiliations:** Department of Epidemiology, Rollins School of Public Health, Emory University, Atlanta, GA 30322, United States; Department of Epidemiology, Rollins School of Public Health, Emory University, Atlanta, GA 30322, United States; Gangarosa Department of Environmental Health, Rollins School of Public Health, Emory University, Atlanta, GA 30322, United States; Department of Paediatrics and Child Health, Red Cross War Memorial Children’s Hospital, University of Cape Town, Cape Town 7700, South Africa; Department of Epidemiology, Rollins School of Public Health, Emory University, Atlanta, GA 30322, United States; Gangarosa Department of Environmental Health, Rollins School of Public Health, Emory University, Atlanta, GA 30322, United States; Neuroscience Institute, University of Cape Town, Cape Town 7700, South Africa; Department of Psychiatry and Mental Health, University of Cape Town, Cape Town 7925, South Africa; Department of Epidemiology, Rollins School of Public Health, Emory University, Atlanta, GA 30322, United States; Gangarosa Department of Environmental Health, Rollins School of Public Health, Emory University, Atlanta, GA 30322, United States; Department of Biostatistics, Rollins School of Public Health, Emory University, Atlanta, GA 30322, United States; Department of Paediatrics and Child Health, Red Cross War Memorial Children’s Hospital, University of Cape Town, Cape Town 7700, South Africa; South African Medical Research Council (SAMRC) Unit on Risk and Resilience in Mental Disorders, University of Cape Town, Cape Town 7700, South Africa; Neuroscience Institute, University of Cape Town, Cape Town 7700, South Africa; Department of Psychiatry and Mental Health, University of Cape Town, Cape Town 7925, South Africa; South African Medical Research Council (SAMRC) Unit on Risk and Resilience in Mental Disorders, University of Cape Town, Cape Town 7700, South Africa; Department of Epidemiology, Rollins School of Public Health, Emory University, Atlanta, GA 30322, United States; Gangarosa Department of Environmental Health, Rollins School of Public Health, Emory University, Atlanta, GA 30322, United States

**Keywords:** air pollution, psychopathology, mixtures, psychosocial factors

## Abstract

Prenatal indoor air pollution and maternal psychosocial factors have been associated with adverse psychopathology. We used environmental-exposure mixture methodology to investigate joint effects of both exposure classes on child behavior trajectories. For 360 children from the South African Drakenstein Child Health Study, we created trajectories of Child Behavior Checklist scores (at 24, 42, and 60 months) using latent-class linear mixed effects models. Indoor air pollutants and psychosocial factors were measured during pregnancy (second trimester). After adjusting for confounding, single-exposure effects (per natural log-1 unit increase) were assessed using polytomous logistic regression models, joint effects using self-organizing maps, and principal component analysis. Three trajectories were chosen for both internalizing and externalizing problems, with “high” (externalizing) or “increasing” (internalizing) being the most adverse trajectories. High externalizing trajectory was associated with increased exposure to particulate matter of ≤ 10 microns in diameter (PM_10_) (odds ratio [OR] = 1.25; 95% CI, 1.01-1.55) and self-organizing maps exposure profile most associated with smoking (OR = 2.67; 95% CI, 1.14-6.27). Medium internalizing trajectory was associated with increased emotional intimate partner violence (OR = 2.66; 95% CI, 1.17-5.57), increasing trajectory with increased benzene (OR = 1.24; 95% CI, 1.02-1.51) and toluene (1.21; 95% CI, 1.02-1.44) and the principal component most correlated with benzene and toluene (OR = 1.25; 95% CI, 1.02-1.54). Prenatal exposure to environmental pollutants and psychosocial factors was associated with internalizing and externalizing child behavior trajectories. Understanding joint effects of adverse exposure mixtures will facilitate targeted interventions to prevent childhood psychopathology.

**This article is part of a Special Collection on Mental Health**.

## Introduction

Childhood psychopathology, including emotional and behavioral problems, affects parents, teachers, and most importantly the child, and can persist into adulthood.^1^ Experiencing mental health problems before age 14 years is associated with increased risk of adult psychopathology. Psychopathology is characteristically split into 2 categories of disorders, internalizing and externalizing. Externalizing behaviors reflect the behavior towards the environment, whereas internalizing behaviors are reflected inward. Externalizing conditions include attention-deficit/hyperactivity disorder and oppositional defiant disorder, and internalizing conditions include anxiety and depression.^2^^,^^3^

Pregnancy is a sensitive period of brain development, as the central nervous system begins to develop as early as the first month of gestation.^4^ Investigating modifiable risk factors of childhood psychopathology during pregnancy has the potential to reduce the burden of mental health problems in both children and adults.^5^

Indoor air pollution is a ubiquitous and well-known contributor to the global burden of disease.^6^^,^^7^ Animal studies have shown that exposure to air pollutants during pregnancy affects the central nervous system of the fetus and elements of behavior in adulthood.^8^^,^^9^ Epidemiologic studies in humans have also shown that exposure to outdoor air pollutants during pregnancy and early life affects child psychopathology.^10^^‑^^12^ A Korean study found that maternal smoking during pregnancy was associated with adverse Childhood Behavior Checklist (CBCL) total problems scores at 5 years.^13^ Additionally, indoor air pollutants from maternal cooking during pregnancy have been associated with hyperactive behaviors in children at 3 years old, although the study did not measure individual air pollutants, and instead used survey information on cooking fuel as a proxy measure.^14^

Exposure to adverse psychosocial factors during pregnancy also negatively affects child development and mental health. Animal studies have shown that prenatal stress affects behavior,^15^^‑^^17^ and there is growing epidemiologic evidence that prenatal psychosocial stressors are associated with impaired child cognitive development.^18^ The Australian Raine study found that stressful life events during pregnancy, family income below poverty line, and mother not finishing high school were associated with increased CBCL T-scores from age 2-14 years.^19^ Additionally, stressful events during pregnancy, including the death of a relative and financial problems, were associated with increased levels of behavior problems in childhood and problematic mental health trajectories in the Raine study.^19^^,^^20^

Childhood psychopathology can be affected by both psychosocial and environmental factors, and joint effects of these exposures are poorly understood,^21^ as they are usually researched separately. Joint effects of environmental and psychosocial factors are probable, and have been demonstrated in previous research on birth outcomes such as birthweight and gestational age.^21^^‑^^24^ It is important to investigate joint effects of environmental and psychosocial factors because such investigation may help to identify especially vulnerable subgroups, in which the disease burden is likely to be larger than in the general population. So far, very few studies have investigated joint effects of air pollution and psychosocial factors on childhood psychopathology. One epidemiologic study conducted in New York, NY, found that prenatal exposure to polyaromatic hydrocarbons increased the effect of exposure to psychosocial stress on CBCL score in school-age children.^11^ Another study, conducted in Boston, MA, found that black carbon exposure during pregnancy was significantly associated with lower attention concentration index scores in boys with high exposure to prenatal stress.^25^ However, both of these studies only used one air pollutant and one psychosocial factor when investigating joint effects of air pollution and psychosocial stress on child psychology instead of an exposure mixture. This does not reflect real-life exposure as people are exposed to many pollutants and psychosocial factors at the same time.^21^ Additionally, both of these studies were conducted in the United States, a high-income country.

A majority of research on childhood psychopathology is conducted in high-income countries, yet almost 80% of children live in low to middle-income countries.^26^ Indoor air pollution is an important source of air pollution in low to middle-income countries, where many homes rely on alternate fuel sources for household energy, and particularly affects women, who through traditional gender norms generally spend more time indoors and are more involved in food preparation and cooking.^6^^,^^27^ Additionally, in low- to middle-income countries, prevalence of perinatal depression and exposure to violence is even higher than in high-income countires.^28^ Therefore, pregnant women in low- to middle-income countries may be uniquely susceptible to the joint effects of indoor air pollution and psychosocial factors.

We aimed to investigate the individual and joint effects of prenatal exposure to indoor air pollution and maternal psychosocial factors on trajectories of psychopathology in early childhood in a South African birth cohort. This study used traditional single-exposure polytomous logistic regression modeling to investigate the effects of indoor air pollutants and psychosocial factors during pregnancy individually, as well as exposure mixture methods, such as self-organizing maps (SOMs), principal components analysis (PCA), and quantile g-computation to investigate joint effects of exposures.

## Methods

### Data source

This study uses data from a subset of participants enrolled in the Drakenstein Child Health Study (DCHS), a multidisciplinary population-based pregnancy cohort based in South Africa. Pregnant women were recruited in their second trimester of pregnancy, from 2012-2015, and follow-up with mother-child pairs has been conducted annually thereafter. Pregnant women seeking care at 2 public sector primary health care clinics, who were at least 18 years of age, were within 20-28 weeks’ gestation, and had no intention of moving away from the district were eligible for enrollment. Recruitment for DCHS has been described elsewhere.^28^^,^^29^ The full cohort includes *n* = 1141 mother-child pairs, among whom a subset (*n* = 819) were selected for indoor air pollution measurement. Mother-child pairs with indoor air pollution measurements as well as complete Child Behavior Checklist measurements at 24, 42, and 60 months of age were included in this analysis (*n* = 360) (Table S1). The DCHS was approved by the Human Research Ethics Committee of the Faculty of Health Sciences, University of Cape Town, by Stellenbosch University and the Western Cape Provincial Research committee. Written informed consent was provided by each mother for herself and her child and was renewed annually.

### Indoor air pollution assessment

Indoor air pollution measurements were taken during participants’ second trimester of pregnancy. Pollutants measured include particulate matter of ≤ 10 microns in diameter (PM_10_), carbon monoxide (CO), nitrogen dioxide (NO_2_), sulfur dioxide (SO_2_), and volatile organic compounds (VOCs) benzene and toluene. PM_10_ was collected over 24 hours with a personal air-sampling pump (AirChek 52, SKC Inc.), using a gravimetrically preweighted filter. Carbon monoxide (CO) was collected over 24 hours using an Altair carbon monoxide single gas detection unit; electrochemical sensor detection of gas at 10-minute intervals was collected. Sulfur dioxide (SO_2_) and nitrogen dioxide (NO_2_) were collected over 2 weeks using Radiello absorbent filters in polyethylene diffusive body. VOCs, including benzene and toluene, were collected over 2 weeks using Markes thermal desorption tubes.^30^ Information on type of home, distance from major road, size of home, number of inhabitants, access to basic amenities, fuels used for cooking and heating, ventilation within homes, and pesticides and cleaning materials used in the home was collected at home visits.^30^

### Assessment of psychosocial factors

Psychosocial factors were collected via questionnaire in the second trimester of pregnancy and included multiple dimensions of psychosocial stress. Employment, education, household income, household assets, marital status, number of dependents, and financial activities were included as indicators of socioeconomic status (SES). Perceived household food insecurity was assessed using an adapted version of the US Department of Agriculture Household Food Security Scale.^31^ Intimate partner violence was assessed using the Intimate Partner Violence Questionnaire adapted from the World Health Organization (WHO) multiple-country study and the Women’s Health Study in Zimbabwe.^32^^,^^33^ The Intimate Partner Violence Questionnaire assesses lifetime and recent (previous year) exposure to emotional, physical, and sexual violence. The World Mental Health Life Events Questionnaire (LEQ) was used to measure trauma and resilience. Use of alcohol and tobacco was assessed using the Alcohol, Smoking, and Substance Involvement Screening Test (ASSIST). Additionally, tobacco smoke exposure was assessed via urinary cotinine and questionnaire. The Self Reporting Questionnaire (SRQ-20), a measure endorsed by the WHO, was used to measure psychological distress.^34^^,^^35^ The Edinburgh Postnatal Depression Scale (EPDS) was used to measure depressive symptoms.^36^

### Outcome assessment

Parent-reported child psychopathology was assessed using the preschool version of the CBCL administered at ages 24, 42, and 60 months.^37^ Child behavior was assessed using a 3-point Likert scale (0 = not true; 2 = often or very true) to create a score, consisting of 113 questions, which can be divided into internalizing and externalizing subscores. The internalizing scale combines the scores from the anxious/depressed, withdrawn/depressed, and somatic complaints syndromic scales. The externalizing scale combines the rule-breaking and aggressive behavior syndromic scales.^37^ Higher CBCL scores indicate increased problematic behavior, indicative of child psychopathology. CBCL scores show good associations with psychopathology diagnoses from the *Diagnostic and Statistical Manual of Mental Disorders*, *Fifth Edition*, including, anxiety, oppositional defiant disorder, attention deficit/hyperactivity disorder, among others.^38^ Standardized CBCL scores, or T-scores, for externalizing and internalizing behavior were used as outcomes in our analyses.

### Statistical analysis

#### Multiple imputation of missing values

While there were no missing values in any of the outcome variables or covariates in our final analysis sample, some participants are missing indoor air pollution or psychosocial measurements (Table S2). We assumed these missing exposure variables are missing at random based on inspections of missingness patterns (Figure S1). To increase the sample size, we used multiple imputation to impute these missing exposure values. Using the R (R Foundation for Statistical Computing) package *Hmisc*, indoor air pollution and psychosocial factor variables were imputed using predictive mean matching, with models that include indoor air pollutants, house characteristics, and psychosocial factor measures. Five seed numbers were created using a random number generator, each seed resulted in its own set of multiply imputed variables. One imputed set, from the 5 sets, from each seed was randomly chosen to use for analyses. The seed with the highest *R*^2^ values, a measure used to explain how well the missing variable was predicted, was selected for primary analysis. *R*^2^ values for each multiple imputed exposure variable differed between seeds. Analyses using complete cases and the other imputation seeds were conducted as a sensitivity analysis.

#### Assessment of CBCL trajectories

The outcome used in this study was trajectory of CBCL score from children aged 24, 42, and 60 months. Participants with CBCL measurements at all time points (*n* = 360) were included. Trajectories were created using latent class linear mixed effects models (LCMMs). LCMM models for log-transformed CBCL T-scores were used to create latent classes, using child sex as a fixed effect covariate and age in months at CBCL measurement as a random effect covariate. Using the R package *lcmm*, LCMM defines a number of “typical” trajectories of CBCL scores, which then were assigned to participants and used as the outcome in the subsequent polytomous logistic regression analyses (described below).^39^ One to 5 latent classes were evaluated in LCMM models, the final model was selected based on measures of model fit such as Akaike information criterion (AIC), Bayesian information criterion (BIC), sample-size-adjusted BIC (SABIC), and entropy (Table S3). Trajectories for CBCL externalizing and internalizing subscales were created.

#### Single-exposure models

Adjusted polytomous logistic regression models were used to estimate single air pollutant and psychosocial factor effects on CBCL trajectory. Confounding was assessed using directed acyclic graphs informed by prior research and literature reviews (Figure S2). To control for confounding, each model adjusted for maternal age at baseline, maternal HIV status, child ancestry, and SES (when not used as the exposure of interest). In individual models, all exposures were natural log–transformed for modeling. To account for confounding by psychosocial factors, models with indoor air pollutants as the main exposure additionally adjusted for psychosocial factors, and vice versa. The model estimating the effect of SES adjusted for air pollution exposures. To avoid oversaturation of the linear regression models, and as the exposures within each exposure group (air pollution exposure and psychosocial factors) were highly correlated, the first principal components (PCs) of each group were used as confounders instead of the original variables. To create these principal components, we conducted a PCA for each exposure group separately (more details can be found in Appendix S1). In a sensitivity analysis to account for seasonality of indoor air pollution measurement, we adjusted the air pollution exposure models for season of indoor air pollution measurement along with previously mentioned confounders.

#### Joint effects models

We used 2 complementary mixture methods to examine joint effects of indoor air pollution and psychosocial factors: PCA and SOMs. While PCA was not developed as an exposure mixture method, using the first few PCs explains a percentage of the total variance in the data using a smaller number of variables. In addition, PCs are continuous, orthogonal, and uncorrelated, which can increase the statistical power to detect associations in comparison with categorical variables. PCs were calculated based on centered and scaled indoor air pollution and psychosocial exposure variables. PCs of the exposure mixture (combination of indoor air pollution and psychosocial exposure variables) were created and added as exposure variables to the polytomous logistic regression model to investigate the joint effect of indoor air pollution and psychosocial factors on CBCL trajectories. The number of PCs added to the model was determined by proportion of variance contributed as seen in an “elbow plot” (Figure S3), resulting in 5 PCs that explained 55% of the total variance. Polytomous logistic regression models were adjusted for maternal age at baseline, maternal HIV status, and child ancestry.

SOM was used to examine the effects of certain exposure profiles on CBCL trajectory. The SOM algorithm identifies exposure cluster profiles with exposure levels homogenous within the cluster and heterogeneous between clusters.^40^ The advantage of SOM over PCA is interpretability of the exposure profile clusters. However, with smaller sample sizes there can be low numbers of participants in some clusters. The number of clusters chosen for analysis was based on multiple statistical measures identifying group structure, including AIC and adjusted *R*^2^ as well as visual inspection of the clusters for interpretability and suitable number of participants in each cluster, resulting in 4 SOM clusters to represent prenatal exposure profiles (Table S4). Indoor air pollution and psychosocial factor variables were centered and scaled before running the SOM clustering algorithm. The effect of these exposure clusters on CBCL trajectory was assessed using adjusted polytomous logistic regression. Models adjusted for maternal age, maternal HIV status, and child ancestry.

Finally, we used quantile g-computation to estimate the overall effect of our exposure mixture on a dichotomized version of the CBCL trajectories.^41^ Quantile g-computation cannot analyze a 3-level outcome variable, so high and medium trajectories were combined and contrasted with low trajectories. Using the *qgcomp* R package, an overall mixture effect and partial effect contributions from each exposure were calculated for high/medium vs low CBCL trajectory adjusted for maternal age, maternal HIV status, and ancestry. The full model was fitted using binomial regression and fitted for 10 deciles of exposure and 200 bootstrapped samples. All analyses were performed using R version 3.6.1 (R Core Team, Vienna, Austria).

## Results

### Study population

The final sample for this analysis consisted of 360 mother-child pairs. The mean maternal age during pregnancy was 26.9 (SD = 5.6) years. Nearly a quarter of mothers (*n* = 78; 21.7%) were HIV-positive at baseline. Half of the children were male (*n* = 190; 52.8%). In this sample 50.3% (*n* = 181) of mothers identified their children as having mixed ancestry, the other half identified as having Black African ancestry (*n* = 179; 49.7%) (Table 1). Table S1 compares demographic and exposure characteristics in the full cohort, the indoor air pollution subsample, and the analytical sample. Demographic characteristics and psychosocial factor scores are similar across samples. Indoor air pollutant exposure concentrations in the analysis sample are slightly lower than in the full cohort, except for PM_10_ (analysis sample median: 41.46 μg/m^3^ vs full cohort: 33.45 μg/m^3^) and cotinine (analysis sample median: 61.34 ng/mL vs full cohort: 43.00 ng/mL) which are higher than in the full cohort.

**Table 1:** Population characteristics (*n* = 360), Drakenstein Child Health Study, South Africa, 2012-2015.

**Characteristic**	**Summary statistic**
Maternal age, years^a^	26.87 (5.6)
Male child, %^a^	190 (52.8)
Child ancestry, %^a^	
Black African	179 (49.7)
Mixed Ancestry	181 (50.3)
Mother HIV-positive, %^a^	78 (21.7)
CBCL externalizing trajectory, %^a^	
1 (medium)	175 (48.6)
2 (low)	80 (22.2)
3 (high)	105 (29.2)
CBCL internalizing trajectory, %^a^	
1 (increasing)	89 (24.7)
2 (decreasing)	158 (43.9)
3 (medium)	113 (31.4)
PM_10_ μg /m^3^^b^	40.72 (14.03-69.07)
CO mg/m^3^^b^	0.00 (0.00-60.00)
Benzene μg/m^3^^b^	3.28 (1.11-8.63)
Toluene μg/m^3^^b^	15.42 (5.93-42.94)
NO_2_ μg/m^3^^b^	5.90 (2.66-11.14)
SO_2_ μg/m^3^^b^	0.00 (0.00-0.18)
Urine cotinine ng/ml^b^	58.95 (13.67-500.00)
SES asset sum^b^	7.00 (5.00-8.00)
Food insecurity total score^b^	0.00 (0.00-2.00)
SRQ-20 total score^b^	4.00 (1.00-7.00)
EPDS total score^b^	9.00 (6.00-12.00)
LEQ total score^b^	1.00 (0.00-3.00)
Emotional IPV score^b^	5.00 (4.00-7.00)
Physical IPV score^b^	6.00 (5.00-8.00)
ASSIST tobacco score^b^	0.00 (0.00-18.00)
ASSIST alcohol score^b^	0.00 (0.00-0.00)

Abbreviations: ASSIST, Alcohol, Smoking, and Substance Involvement Screening Test; CBCL, Childhood Behavior Checklist; CO, carbon monoxide; EPDS, Edinburgh Postnatal Depression Scale; IPV, intimate partner violence; LEQ, Life Experiences Questionnaire; NO_2_, nitrogen dioxide; PM_10_, particulate matter of ≤ 10 microns in diameter; SES, socioeconomic status; SO_2_, sulfur dioxide; SRQ-20, Self-Reporting Questionnaire.

^a^Values reported as mean (standard deviation).

^b^Values reported as median (interquartile range).

### Trajectories

At each time period, CBCL internalizing and externalizing subscores were highly correlated (24 months: Pearson *r* = 0.71; 42 months: *r* = 0.7; 60 months: *r* = 0.82; Table S5).

**Figure 1:**
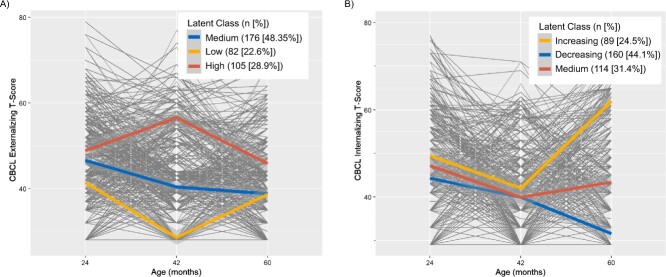
Latent class mixed model (LCMM) trajectories. Child Behavior Checklist (CBCL) T-score trajectories modeled using LCMM, adjusted for child sex and age in months at CBCL assessment in the Drakenstein Child Health Study, South Africa, 2012-2015. A) CBCL externalizing-problems T-score; B) CBCL internalizing-problems T-score.

Three trajectories were chosen for CBCL externalizing problems and internalizing problems (Table S3). Trajectories for externalizing problems were categorized as “high,” “medium,” and “low” trajectories, as they correspond to relatively high, medium, and low scores across time points (Figure 1A, Table S6). Latent class 1, the “medium” trajectory decreased slightly over time but scores were always between the “high” and “low” trajectory. Latent class 2 had the lowest CBCL externalizing scores over the study period. In contrast, latent class 3 always had the highest CBCL externalizing score over the study period. In all regression analyses the “low” trajectory was used as the reference group.

CBCL internalizing problems score trajectories were categorized as, having “decreasing,” “medium,” and “increasing” trajectories., For the internalizing problems scores, all 3 trajectories slightly decreased between 24 and 42 months and diverged from 42 to 60 months (Figure 1B, Table S6). The CBCL internalizing-problems trajectory shown in latent class 1 is characterized as having a sharply increasing CBCL internalizing score after 42 months, this trajectory is described as the “increasing” trajectory. In contrast, latent class 2 shows a decreasing trajectory over the time period and will be described as the “decreasing” trajectory. Latent class 3 is stable over time and scores are between the increasing and decreasing trajectories, and will therefore be described as the “medium” trajectory. In all regression analyses the “decreasing” trajectory was used as the reference group.

### Single-exposure models

**Figure 2:**
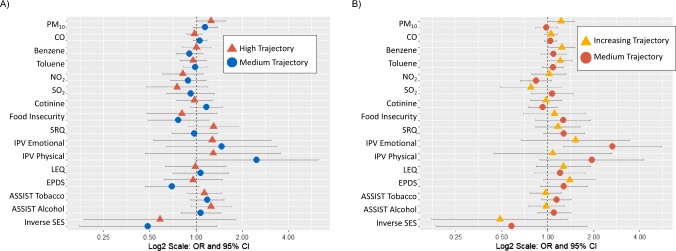
Results from single-exposure polytomous logistic regression models, adjusted for maternal age, maternal HIV status, and ancestry (*n* = 360), Drakenstein Child Health Study, South Africa, 2012-2015. A) Child Behavior Checklist (CBCL) externalizing problems; B) CBCL internalizing problems. Odds ratios (ORs) are presented on a log2 scale to improve readability of effect estimates. ASSIST, Alcohol, Smoking, and Substance Involvement Screening Test; CO, carbon monoxide; EPDS, Edinburgh Postnatal Depression Scale; LEQ, Life Experiences Questionnaire; NO_2_, nitrogen dioxide; PM_10_, particulate matter of ≤ 10 microns in diameter; SES, socioeconomic status; SO_2_, sulfur dioxide; SRQ-20, Self-Reporting Questionnaire.

PM_10_ was associated with high externalizing-problems trajectory (odds ratio [OR] = 1.25; 95% CI, 1.01-1.55) (Figure 2A, Table S7). Benzene and toluene were associated with increasing internalizing-problems trajectory (for benzene, OR = 1.24; 95% CI, 1.02-1.51; and for toluene, OR = 1.21; 95% CI, 1.02-1.44). Emotional intimate partner violence score was associated with higher odds of the medium internalizing trajectory (OR = 2.66; 95% CI, 1.27-5.57) (Figure 2B, Table S7).

### Joint effects models

**Figure 3:**
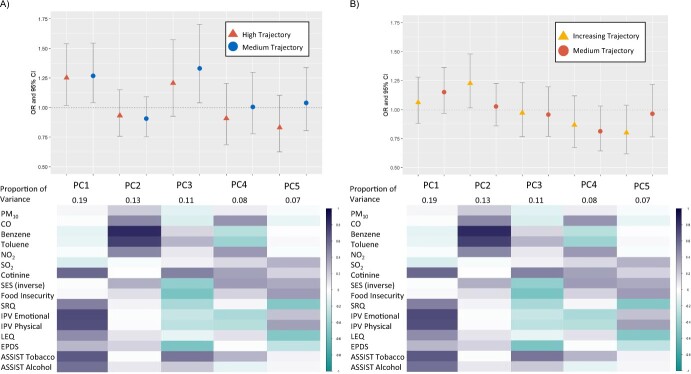
Top panels show results of polytomous logistic regression modeling using principal components of exposure mixture, adjusted for maternal age, maternal HIV status, ancestry in the Drakenstein Child Health Study, South Africa, 2012-2015. Bottom panels show a correlation matrix between individual air pollutant and psychosocial factor exposures and each principal component. Purple indicates higher positive correlation, while teal indicates higher negative correlation. A) Child Behavior Checklist (CBCL) externalizing problems; B) CBCL internalizing problems. ASSIST, Alcohol, Smoking, and Substance Involvement Screening Test; CO, carbon monoxide; EPDS, Edinburgh Postnatal Depression Scale; LEQ, Life Experiences Questionnaire; NO_2_, nitrogen dioxide; OR, odds ratio; PC, principal component; PM_10_, particulate matter of ≤ 10 microns in diameter; SES, socioeconomic status; SO_2_, sulfur dioxide; SRQ-20, Self-Reporting Questionnaire.

The 5 PCs selected to be included in joint effects modeling have differential loadings that represent directions of correlations in the data (Figure 3). PC1 is explained by high positive correlation with adverse psychosocial factors such as cotinine level, intimate partner violence, and ASSIST tobacco and alcohol scores. PC2 is uncorrelated with psychosocial factors, and is characterized by increased indoor air pollution, particularly benzene and toluene. PC3 is negatively correlated with most adverse psychosocial factors, except for a positive correlation with smoking and alcohol. PC4 is characterized primarily by high CO and NO_2_ and low SES. PC5 is characterized by low SES, high food insecurity, and intimate partner violence. In adjusted polytomous logistic regression models using externalizing CBCL trajectory, PC1 was associated with both high (OR = 1.25; 95% CI, 1.02-1.54) and medium (OR = 1.27; 95% CI, 1.04-1.54) trajectories and PC3 was significantly associated with the medium (OR = 1.33; 95% CI, 1.04-171) trajectory compared with the low trajectory. Both PC1 and PC3 are explained by high cotinine level and high ASSIST Tobacco and Alcohol scores. The more robust association with PC1 reflects that externalizing CBCL trajectory is associated with both smoking-related exposures and high psychosocial stressors (Figure 3A, Table S8). Increasing internalizing CBCL trajectory was associated with PC2 (OR = 1.22; 95% CI, 1.02-1.48), the PC mostly explained by high benzene and toluene levels in adjusted polytomous regression models (Figure 3B, Table S8).

The 4 clusters identified by the SOM algorithm represent exposure profiles seen in this population. Participants with a cluster 1 exposure profile have relatively low exposure to all environmental and adverse psychosocial exposures, while participants with a cluster 2 exposure profile have high exposure to indoor air pollutants, low SES, and depression. The cluster 3 exposure profile represents participants with smoking and alcohol use. Finally, the cluster 4 exposure profile has high exposure to most adverse psychosocial factors and PM_10_. In SOM analyses with an externalizing-problems trajectory, the cluster associated with high cotinine level and ASSIST tobacco and alcohol scores (cluster 3), indicative of tobacco and alcohol use, was associated with the high trajectory (OR = 2.67; 95% CI, 1.14-6.27), compared with the low-exposure cluster (cluster 1; Figure S4, Table S9), in line with the PCA analysis. No SOM exposure cluster was associated with CBCL internalizing-problems trajectories.

In quantile g-computation models, the overall mixture was not significantly associated with high/medium compared with low externalizing (mixture OR = 1.42; *P* = 0.50) or medium/increasing internalizing (mixture OR = 1.59; *P* = 0.29) trajectories. Although not statistically significant, there is a slight positive trend in odds of both high/medium externalizing and medium/increasing internalizing trajectory as deciles of the exposure mixture increase (Figure S5).

### Sensitivity analyses

Results from sensitivity analysis models comparing complete cases and imputed exposures were similar to the main results presented above. In the internalizing-problems models, toluene was also associated with the increasing trajectory in one other imputation model, but ORs were consistent across all models. Similarly for benzene, the association with increasing trajectory was not significant for other models, but the ORs were consistent across all models (Table S7). Results for sensitivity analyses for single-exposure models for air pollution adding season of air pollution measurement as a confounder were also similar to the main results presented above (Table S10).

## Discussion

In this analysis of mother-child pairs from a South African birth cohort, trajectories of internalizing and externalizing child behavior at 24, 42, and 60 months were differentially associated with prenatal indoor air pollution and psychosocial exposures. The internalizing-problems trajectory was individually associated with both exposures to indoor air pollution and adverse psychosocial factors, while the externalizing-problems trajectory was mostly associated with smoking-related exposures. This analysis indicates that different exposures might differently affect internalizing and externalizing problem trajectories.

We observed that the externalizing-problems trajectory was most associated with prenatal smoking behaviors and PM_10_, a by-product of cigarette smoke. This has been observed in a previous study from the DCHS^42^. In other prior studies investigating PM_10_ exposure during pregnancy and autism spectrum disorder, a condition that exhibits externalizing behaviors, results have been mixed. Only one study found a significant harmful effect of PM_10_^43^; the rest found a null association.^44^ Other studies investigating smoking during pregnancy have found associations with externalizing behaviors, including inattention and impulsivity.^45^^‑^^47^

The internalizing-problems trajectory was associated with emotional intimate partner violence. Few studies have investigated the association between intimate partner violence before and during pregnancy and childhood psychopathology. One study found that intimate partner violence during pregnancy was correlated with borderline or clinical internalizing problems, externalizing problems, and total problems using the CBCL in children aged 18 months to 18 years.^48^ However, results from our study may not be directly comparable as that study did not separate different types of intimate partner violence and did not adjust for confounding. More epidemiology studies investigating prenatal exposure to intimate partner violence and their association with child behavior are needed. Our study also found individual and joint associations with VOCs, specifically benzene and toluene, and internalizing-problems trajectory.

To our knowledge, our study is the first to investigate the association between prenatal VOC exposure and childhood psychopathology. One prior study has investigated VOCs and neurodevelopmental outcomes, although they measured exposure in early childhood, and found that m,p-xylene and o-xylene were associated with decreased scores in the Ages and Stages Questionnaire, which screens young children for developmental delays.^49^ Continued epidemiologic research on prenatal VOC exposure is necessary to examine the relationship between VOCs and child psychopathy.

In analyses using PCs and SOM, high air pollution exposure coupled with low SES was associated with internalizing behavior trajectories. This finding indicates that pregnant women with low SES could benefit from indoor-air-pollution interventions to reduce childhood psychopathology. In support of our findings, prior studies investigating neurodevelopmental or psychopathological outcomes at one time period found an interaction between air pollutants and adverse psychosocial factors. However, these studies did not use environmental mixture methods and instead used terms for interaction between one air pollutant and one psychosocial factor.^11^^,^^25^^,^^50^^,^^51^ Studies using other health outcomes have also found joint effects of prenatal air pollution and psychosocial factor exposure. A recent review article found several studies showing prenatal psychosocial factors modifying the effect of ambient and traffic-related air pollution on adverse birth and childhood outcomes such as birthweight, gestational age, and asthma.^21^

Synergy is probable because air pollutants and stress caused by psychosocial factors may impact similar brain mechanisms, including inflammation.^9^^,^^52^ It is hypothesized that air pollution affects the central nervous system via neuroinflammation and oxidative stress.^9^^,^^53^^‑^^56^ Animal models show that air pollutants cause a systemic inflammatory response, including neuroinflammation inside the brain. Both the physical air pollutant particle, and the toxic components absorbed on the particle can create an inflammatory response. Translocation of air pollutant nanoparticles from the lungs and nasal pathways to other areas of the body, including the placenta,^57^ cause damage to the mother’s body and fetus. Microglia respond to this damage by releasing inflammatory cytokines like tumor necrosis factor–α, interleukin-1β, and interleukin-6, and reactive oxygen species (ROS), inducing oxidative stress. Chronic activation of the microglia and overproduction of inflammatory markers and ROS can cause neuronal cell death.^9^ A range of research also documents that adverse psychosocial factors are associated with neuroinflammation and oxidative stress.^52^^,^^56^^,^^58^

### Strengths and limitations

Several strengths of this study deserve emphasis. First, this analysis used measurements of multiple indoor air pollution exposures. Indoor air pollution in low- and middle-income countries is most often measured using survey-based proxy measures, eg, cooking practices, smoking, etc. Given the well-known harmful effects of indoor air pollution,^27^ it is important to measure individual pollutants to pinpoint which chemical, or combination of chemicals, is causing health problems and the biological mechanisms involved. Second, this study leverages a unique prospective birth cohort with repeated measurements of psychopathology across time periods. And finally, the traditional single-exposure analysis is complemented with 2 environmental mixtures methods, PCA and SOM. These methods allow us to explore joint effects of environmental and social exposure profiles that are associated with psychopathology trajectory. Estimating joint effects can identify vulnerable subgroups to target for interventions to reduce childhood psychopathology.

Several limitations deserve emphasis. First, we were unable to investigate the effect of the total mixture on trajectories or psychopathology. Currently, environmental mixture methods that estimate a total mixture effect (eg, Bayesian kernel machine regression (BKMR), weighted quantile sum regression, etc.) can only accommodate linear or logistic regression, and not polytomous logistic regression. We created a dichotomized trajectory variable (high/medium vs low and increasing/medium vs decreasing) to estimate an overall mixture effect using quantile g-computation; however, collapsing trajectory categories led to a loss of information, which consequently reduced the statistical power to detect associations with the overall mixture. An additional limitation is that we were unable to investigate interaction between exposures (synergy/antagonism). Alternative methods like BKMR can estimate interactions between exposures on binary or continuous outcomes. However, BKMR works best with a smaller number of exposures and a larger sample size, especially if the goal is to look at pairwise interactions between exposures. In contrast, one advantage of the SOM methods that we used is that the SOM profiles are based on how exposures co-occur in real life. These profiles can be used for more practical interventions (eg, highlighting vulnerable subgroups of people who are exposed to both air pollution and depression) than looking at pairwise synergy/antagonism, which may not correspond to exposures that appear in real life. As methods to investigate environmental exposure mixtures advance this may be an option for future studies Second, the lack of measurements of fine (PM_2.5_) and ultrafine PM; PM_2.5_ measurement was not collected because, at the time, personal PM_2.5_ monitoring was not easily available for this large a study. Additionally, this study uses indoor-air-pollution measurements from one point (24 hours or 2-week average, depending on pollutant) in the second trimester and in early life to characterize exposure for the whole period. Psychosocial measures were administered at one time in the second trimester. This could create some misclassification of the exposure by either over- or underestimating a participant’s exposure during pregnancy. Furthermore, by collecting exposure data only in the second trimester of pregnancy, we may be missing important effects of pollutants and psychosocial factors in early pregnancy, or effects of exposure more proximal to the outcome. Nevertheless, prior research in the DCHS has found associations between exposures in the second trimester and several outcomes,^57^^,^^58^ including neurodevelopment.^59^ Other limitations include biases from residual confounding and selection bias, although this cohort was selected to be population based and representative of periurban populations in South Africa and other low-income countries. While this study had a relatively small sample size, few studies have such detailed exposure information and repeated outcomes, especially in a low to middle-income country.

### Conclusion

We observed single-exposure and joint effects of prenatal exposure to indoor air pollutants and psychosocial factors on trajectories of childhood psychopathology in children aged 24-60 months from South Africa. Externalizing and internalizing trajectories were associated with prenatal indoor air pollution and psychosocial factors. Investigating joint effects of environmental and social exposures is necessary for identifying vulnerable subgroups to target for interventions to reduce exposure and burden of childhood psychopathology.

## Supplementary Material

Web_Material_kwae046

## Data Availability

Data may be available upon request from H.J.Z. (heather.zar@uct.ac.za).
